# Prevalence of late xerostomia and hyposalivation with associated risk factors in survivors of head and neck cancer after radiotherapy: a multi-centric cross-sectional study

**DOI:** 10.1186/s13014-025-02737-1

**Published:** 2025-11-07

**Authors:** Asmaa Abou-Bakr, Fatma E. A. Hassanein, Hany William, Suzan S. Ibrahim

**Affiliations:** 1 Oral Medicine and Periodontology, Faculty of Dentistry, Galala University, Suez, Egypt; 2 Oral Medicine, Periodontology, and Oral Diagnosis, Faculty of Dentistry, King Salman International University, El Tur, South Sinai, Egypt; 3 Radiation Oncology, Ahmed Maher Teaching Hospital, Cairo, Egypt; 4 Oral Medicine and Periodontology, Faculty of Dentistry, Ain Shams University, Cairo, Egypt; 5 Faculty of Oral and Dental Medicine, Nahda University in Beni Seuf City, Beni Seuf, Egypt

**Keywords:** Dry mouth, Xerostomia, Hyposalivation, Head and neck, Cancer, Radiotherapy

## Abstract

**Objectives:**

This study aimed to assess the prevalence and risk factors associated with late xerostomia and hyposalivation in head and neck cancer (HNC) patients after radiotherapy (RT).

**Materials and methods:**

An observational, multicentric cross-sectional study was conducted on 260 HNC patients attending various radiation centers for follow up 1-year post-treatment. Clinical assessments included the Subjective Dry Mouth Score (SXI), Clinical Oral Dryness Score (CODS), and Unstimulated Salivary Flow Rate (UWS).

**Results:**

Xerostomia was reported by 78% of patients, with higher severity in those over 50 years (Mean ± SD: 13.53 ± 1.09). Women showed lower salivary flow (UWS: *r* = 0.556, *p* < 0.0001) and higher xerostomia scores (SXI: *r* = 0.337, CODS: *r* = 0.359) than men. Tumor site correlated strongly with xerostomia (SXI: *r* = 0.894, *p* < 0.001), with oral cavity tumors showing more severe effects than nasopharyngeal tumors. Higher RT dose and fraction were negatively associated with UWS (*r* = -0.537, *p* < 0.0001) and positively correlated with SXI (*r* = 0.293) and CODS (*r* = 0.405, *p* < 0.0001). The regression models showed that xerostomia severity is significantly predicted by advanced tumor stage, female gender, older age, and higher radiation dose exposure.

**Conclusions:**

The study reveals a high prevalence of xerostomia and hyposalivation among HNC survivors. Increased xerostomia severity and decreased salivary flow were significantly associated with advanced tumor stage, higher radiation doses, and concurrent chemoradiotherapy.

**Clinical Relevance:**

Understanding risk factors can guide early interventions and personalized management to enhance long-term oral health outcomes.

## Introduction

Head and neck cancers (HNCs) are the eighth most common cancer in the world, accounting for over 444,000 deaths and about 878,000 new cases diagnosed each year [1]. As such, they pose a serious threat to global health. These cancers are highly prevalent in men and make up a significant percentage of cancers in men, especially in areas where risk factors like alcohol and tobacco use are prevalent [2]. In 2035, there will likely be a 62% increase in the incidence of oral cancers [3, 4].

HNC is a highly heterogeneous disease, making its management complex. Effective treatment typically involves a multimodal approach, including surgery followed by radiotherapy, which may be administered alone or in combination with chemotherapy [5, 6]. However, radiation therapy affects both cancerous and normal cells, leading to treatment-related toxicities [7]. These toxicities are categorized as acute if they occur within 90 days of treatment and late if they develop after this period [8].

Acute toxicities of radiotherapy commonly include mucositis, dermatitis, dysphagia, odynophagia, hoarseness, and loss of taste, often resulting from laryngeal edema. In contrast, late toxicities—such as osteoradionecrosis, xerostomia, subcutaneous fibrosis, thyroid dysfunction, trismus, sensorineural hearing loss, myelitis, and pharyngeal or oropharyngeal stenosis—may manifest months or even years after treatment. Typically, tissues with high cell turnover are more susceptible to acute reactions, whereas tissues with slower turnover exhibit late complications [9, 10].

Xerostomia, or the subjective perception of dry mouth [11], is one of the most common and persistent side effects of radiation therapy (RT) and chemoradiotherapy (CRT) in HNC treatment. It results from damage to the salivary glands, leading to reduced or absent saliva production. This condition significantly impacts oral health, causing pain, dysphagia (difficulty swallowing), speech difficulties, altered taste, increased dental decay, infections, and even osteoradionecrosis [12, 13]. Although xerostomia is often associated with low salivary flow (hyposalivation), some individuals may experience dry mouth despite having normal salivary flow rates, indicating that xerostomia is not always a direct reflection of salivary gland dysfunction [14, 15]. Hyposalivation is objectively assessed by measuring salivary flow rates, which are classified as unstimulated (resting) saliva, with a normal range of 0.29–0.41 ml/min, and stimulated saliva, which aids in chewing and digestion, typically ranging between 1 and 2 ml/min [16].

Identified risk factors for salivary gland dysfunction in cancer patients and survivors include head and neck radiotherapy, total body irradiation during hematopoietic stem cell transplantation (HSCT), graft-versus-host disease, and female gender [17]. However, further research is required to explore additional contributing factors. Given the high prevalence of xerostomia and hyposalivation as late side effects in HNC patients, there is a lack of representative studies on this condition among the Egyptian population.

Although the pathophysiology of RT-induced xerostomia is universally well established, institutional and geographical circumstances can considerably affect its prevalence and perceived severity. Due to delayed presentation, patients in the Egyptian healthcare system tend to have advanced tumor stages at diagnosis [18], which can result in larger radiation fields and doses that adversely intensifies the salivary gland damage. The subjective burden of xerostomia and its impact on quality of life may also be exacerbated by cultural and socioeconomic factors, such as intake of high carbohydrate and spice diets, high dehydration rates secondary to hot climates, and limited access to preventive oral care and symptom palliation modalities (e.g., special fluoride treatments, saliva substitutes). With the aim of providing data that might represent issues in comparable resource-variable settings. Comorbidities and impaired overall health burdens tend to worsen these difficulties [19]. This study aims both to measure the burden of late xerostomia and to situate it within Egypt’s distinctive clinical and cultural situation.

Therefore, this study aims to evaluate the prevalence and associated risk factors of late-onset xerostomia based on unstimulated salivary flow rates in patients treated with intensity-modulated radiation therapy (IMRT), with or without concurrent chemotherapy, for HNC.

## Subjects and methods

### Sample size calculation

A power analysis was conducted to ensure adequate statistical power for evaluating the prevalence of xerostomia among long-term oropharyngeal cancer survivors. To achieve this, a 95% confidence interval (CI) and a 5% margin of error were applied, incorporating finite population correction. The estimated prevalence of xerostomia (78.41%) was derived from a previous study^1^ [20]. Based on these parameters, the predicted sample size (n) was determined to be 261 cases. The sample size calculation was performed using R software, version 4.4.1 for Windows.^2^

### Study design

The present study was an observational, quantitative, analytical, multi-centric cross-sectional study, reviewed and approved by the Ahmed Maher Hospital Research Ethics Committee (HAM00221). Before participation, patients were provided with a detailed explanation of the study procedures and were required to sign an informed consent form. To ensure confidentiality, all personal information and study findings were securely stored in password-protected file systems. Additionally, patient names were encoded using a unique coding method known only to the primary investigator, ensuring that identifiable information was not included in the data analysis.

### Aim of the study

This study aimed to evaluate the prevalence of late xerostomia and hyposalivation, along with their associated risk factors, among long-term head and neck cancer survivors one year after completing radiation therapy, with or without concomitant chemotherapy.

### Patients’ selection

Head and neck cancer survivors were selected from multiple centers. To confirm their experience of xerostomia and dry mouth, they were asked the following question prior to study participation, as documented in previous research [20, 21]: *“How often do you feel that your mouth is dry?”* The response options included *“never*,*” “sometimes*,*” “usually*,*”* and *“always.”* Participants who answered *“usually”* or *“always”* were considered to have xerostomia and were included in the study [21].

**Fig. 1:**
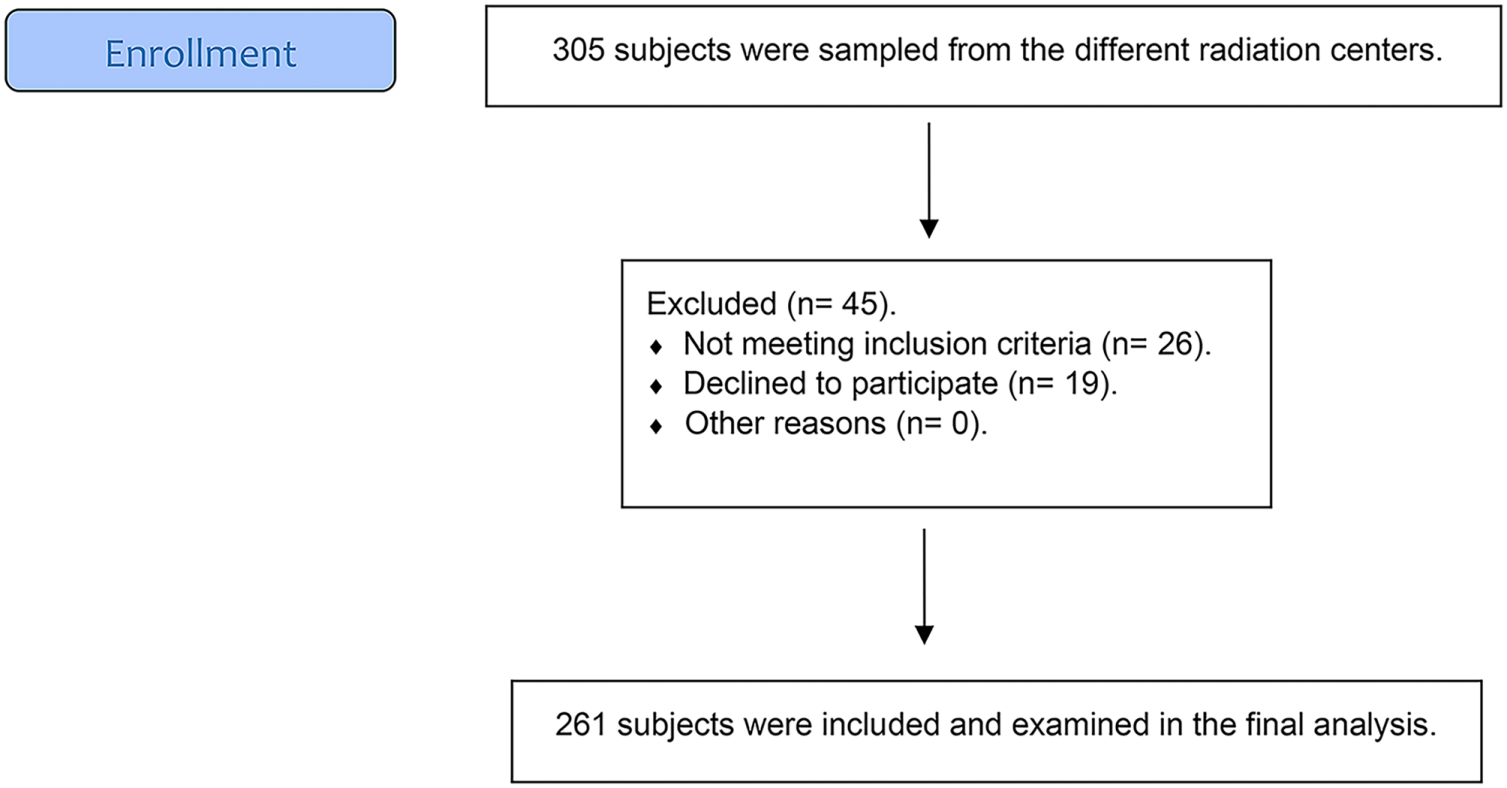
The flow chart for patient’s recruitment

Those who confirmed experiencing xerostomia were further evaluated for eligibility. Inclusion criteria required participants to be men or women aged 18 years or older, willing to undergo the proposed examinations, and having completed curative radiation therapy for head and neck cancer at least one year before survey administration, with or without chemotherapy. Patients with a secondary primary malignancy or recurrent HNC before survey administration were excluded from the study. Eligible HNC survivors then underwent both subjective and objective evaluations for dry mouth, conducted by the investigator (**F.A**). The patient recruitment process is illustrated in Fig. 1.

### Primary outcome

The primary outcome variable of this study was cancer treatment-related xerostomia. Xerostomia was assessed based on the following criteria:

**Subjective dry mouth severity** was measured using the **Summated Xerostomia Inventory (SXI)** [22], which consists of five statements:

1. After a meal, my mouth feels dry.
2. I have a dry mouth.
3. I find it challenging to eat dry meals.
4. I have trouble swallowing some food.
5. My lips look chapped.

Each statement had three response options: *never* (score = 1), *occasionally* (score = 2), and *often* (score = 3). The SXI has a total score range of **5 to 15**, with higher scores indicating more severe dry mouth symptoms. A validated reference suggests that a score of **8** is typical in the general population [23]. Therefore, participants with an SXI score greater than eight were considered positive for subjective xerostomia, while those scoring 8 or below were excluded from further analysis.

### Secondary outcomes

**1. The Clinical Oral Dryness Score (CODS)** is a validated tool used to assess oral dryness based on multiple clinical and visual indicators of the oral cavity [23, 24]. CODS was assessed once during the post-treatment evaluation visit, in line with the study’s cross-sectional design [6, 25].

**The ten features assessed in CODS were**.

1. The mirror adheres to the buccal mucosa.
2. The mirror adheres to the tongue.
3. Slobbering saliva.
4. Absence of a saliva pool on the tongue.
5. Tongue lacking papillae.
6. Altered or refined gingival architecture.
7. Glassy appearance of the palate and other oral mucosa.
8. Lobulated or fissured tongue.
9. Recent or active decay affecting at least two teeth.
10. Presence of debris on the palate (excluding the area beneath dentures).

A higher overall CODS score indicated more severe oral dryness.

**Interpretation of the COD Score** [24].

The **Clinical Oral Dryness Score (CODS)** categorizes oral dryness severity based on an additive scoring system:

- **Mild dryness (score 1–3)**: May not require treatment.
- **Moderate dryness (score 4–6)**: Indicates noticeable dryness that may require monitoring or intervention.
- **Severe dryness (score 7–10)**: Suggests significant clinical dryness, necessitating medical intervention.

**2. Salivary Flow Assessment**: To ensure consistency and accuracy, study personnel from the participating centers were trained in standardized salivary flow assessment procedures. During the clinical visit, unstimulated salivary flow rate (UWS) was measured following internationally recognized protocols. Participants were instructed to first swallow to clear any residual saliva, then spit all accumulated saliva into a plastic container every 30 s for 5 min [26]. Unsimulated salivary flow rates were determined gravimetrically and expressed in mL/min [27]. Hyposalivation was defined as an unstimulated salivary secretion rate of ≤ 0.1 mL/min.

### Clinical and sociodemographic variables

The following clinical and treatment variables were extracted from electronic medical records: T and N categories (American Joint Committee on Cancer, seventh edition), primary tumor subsite, treatment modality, RT dose, mode/type, fractionation schedule, receipt of chemotherapy or surgery, and solid food diet at baseline (used as a surrogate control for pretreatment oral dysfunction/symptoms). Primary head and neck tumor subsites included the tonsils, base of the tongue, glossopharyngeal sulcus, and others (such as the soft palate, pharyngeal wall, and an oropharyngeal site not otherwise specified). Primary tumor T categories were classified as T1, T2, T3, and T4 [22]. Systemic therapy/chemotherapy was recorded as a yes/no indicator, reflecting the use of any chemotherapy, including induction, concurrent, or adjuvant therapy.

Educational level was categorized as low, middle, or high education. Cigarette smoking status was determined based on the following criteria: participants who had not smoked 100 cigarettes in their lifetime were classified as never smokers; those who had quit more than six months before diagnosis were considered former smokers at the time of diagnosis; and current smokers at diagnosis were further categorized into those who quit subsequently and those who continued smoking [28].

#### Statistical analysis

Categorical and ordinal data were presented as frequencies and percentages, while numerical data were summarized using mean, standard deviation (SD), median, and interquartile range (IQR) values. Normality was assessed by examining the data distribution and performing the Shapiro-Wilk test, which indicated that the data were non-parametric. Associations were analyzed using the Kruskal-Wallis test, followed by Dunn’s post hoc test for pairwise comparisons. Correlations were evaluated using Spearman’s rank-order correlation coefficient. Stepwise binary logistic regression models were used to explore the relationship between recession severity classes and various risk factors. Model selection was based on the Akaike Information Criterion (AIC), with the model having the lowest AIC value chosen for analysis. Leave-one-out cross-validation (LOOCV) was performed before analysis to ensure the generalizability of the results. Linearity in the log odds was assessed using binned residual plots, confirming no significant deviations from linearity. Multicollinearity was evaluated using the Variance Inflation Factor (VIF), ensuring that all predictors had VIF values below 5. The significance level for all tests was set at *p* < 0.05. Statistical analyses were conducted using R statistical analysis software, version 4.4.2 for Windows.^3^

## Results

### Sociodemographic characteristics and clinical variables of the participants

The current study included a total of 261 head and neck cancer (HNC) patients, with the majority (62%) aged between 50 and 60 years and a mean age of approximately 50.9 years. Gender distribution showed a predominance of males, with 162 (62%) male participants compared to 99 (38%) females. Educational levels were primarily categorized as middle (51%), followed by high (24%) and low (25%). Among medical conditions, diabetes was the most prevalent, affecting 33% of the participants, followed by hypertension at 26%. Regarding the family history of cancer, 28% of patients reported a positive family history, whereas 72% had no known family history of cancer, as shown in Table 1.

**Table 1:** Demographic data

Parameter			All patients with HNC (*n* = 261)			Xerostomia (*n* = 204)
**1-Gender [n (%)]**	**Male**		162 (62%)		109(53%)	
	**Female**		99 (38%)		95(47%)	
**2-Age**	**< 50**		110 (38%)		72(35%)	
	**> 50**		161 (62%)		132(65%)	
	**Mean ± SD.**		50.9 ± 8.9		51.9 ± 8	
	**Median (IQR)**		54 (30–60)		55(35–60)	
**3- Educational Level**	**High**		63 (24%)		53(26%)	
	**Middle**		134 (51%)		98(8%)	
	**Low**		64 (25%)		53(26%)	
**4- Medical condition [n (%)]**	**Free**		32(12%)		23(11%)	
	**Compromised**		229(88%)		181(89%)	
	**1**	Hypertension	67(26%)		54(26%)	
	**2**	Hypothyroid	15(6%)		14(7%)	
	**3**	free	32(12%)		23(11%)	
	**4**	Leukemia	28(11%)		24(12%)	
	**5**	Diabetes	87(33%)		64(31%)	
	**6**	Hypertension & Diabetes	25(10%)		19(9%)	
	**7**	Cardiovascular disease	7(3%)		6(3%)	
**5-Cancer family history [n (%)]**	**Positive**		74(28%)		57(28%)	
	**Negative**		187(72%)		147(72%)	

### Tumor and medical characteristics for cancer patients

The primary tumor sites included the oral cavity (54%), larynx (22%), and oropharynx (20%). In terms of cancer staging, T2 was the most common stage, accounting for 50% of cases, with corresponding N-stages showing 43% at N2. Histologically, grade II was the most predominant, observed in 44% of patients. Additionally, a significant majority of patients (69%) did not receive chemoradiotherapy (CRT), as shown in Table 2.

**Table 2:** Medical parameters for HNC patients

Parameter		All patients with HNC (*n* = 261) (*n* = 261)	Xerostomia (*n* = 204)
**7-Primary tumor [n (%)]**	**Larynx**	57(23%)	0
	**Oropharynx**	51(20%)	51(25%)
	**Nasopharynx**	11(4%)	11(5%)
	**Oral cavity**	142(54%)	142(70%)
**8-T-stage [n (%)]**	**T1**	94(36%)	64(31%)
	**T2**	131(50%)	105(51%)
	**T3**	36(14%)	35(17%)
**9-N-stage [n (%)]**	**N0**	46(18%)	28(14%)
	**N1**	103(39%)	83(41%)
	**N2**	112(43%)	93(46%)
**10-Histopathology stage [n (%)]**	**Grade (I)**	71(27%)	53(15%)
	**Grade (II)**	115(44%)	85(42%)
	**Grade (III)**	43(16%)	35(17%)
	**Grade (IV)**	32(12%)	31(15%)
**11-Radiotherapy total dose**	**Mean ± SD**	6420.31 ± 489.99	6496 ± 394
	**Median (IQR)**	6600(5000–7000)	6600(5000–7000)
**12-Radiotherapy fraction**	**Mean ± SD**	31.87 ± 2.64	32.2 ± 2.26
	**Median (IQR)**	33(25–35)	33(25–35)
**13-Chemoradiotherapy [n (%)]**	**No**	80(31%)	70(34%)
	**Yes**	181(69%)	134(66%)

### Prevalence of xerostomia

Among the 261 HNC patients, 204 individuals (78%) experienced xerostomia, as defined by an SXI score of ≥ 9, a CODS score between 0 and 10, or a UWS of ≤ 0.4 ml. This finding highlights a high burden of xerostomia among this population, as shown in Table 3.

**Table 3:** Salivary parameters

Parameter (*n* = 261)		Value
14- Subjective Xerostomia Index (SXI),	**Normal < 9**	57(22%)
	**Xerostomia ≥ 9**	204(78%)
15- Clinical Oral Dryness Score (CODS)	**Normal = 0**	57(22%)
	**Xerostomia (0–10)**	204(78%)
16-Unstimulated whole saliva flow (UWS)	**Normal >0.4 ml**	57(22%)
	**Xerostomia < 0.4ml**	204(78%)

### Salivary variables of HNC patients with xerostomia

Regarding the SXI and CODS scores related to xerostomia, both measures indicated a prevalence of 78%. The SXI scores, with a mean ± SD of **12.92 ± 1.55** and a median of **13**, suggest moderate to severe subjective xerostomia. Similarly, CODS scores, with a mean ± SD of **8.72 ± 1.07** and a median of **9**, indicate noticeable clinical oral dryness. Additionally, the UWS measurements, with a mean ± SD of **0.21 ± 0.09 mL** and a median of **0.2 mL**, demonstrate a significant reduction in unstimulated saliva production. These findings, presented in Table 4, highlight the strong agreement between subjective perceptions of dryness and clinical measurements.

**Table 4:** Prevalence and severity of xerostomia / hyposalivation

Parameter (*n* = 189)				Value	
Xerostomia [n (%)] (*n* = 204) 78%	**No**		57 (22%)		
	**Yes**		204(78%)		
Xerostomia [n (%)] (*n* = 204) 78%	**SXI**	**Mean ± SD.**	12.92 ± 1.55		
		**Median (IQR)**	13(9–15)		
	**CODS**	**Mean ± SD.**	8.72 ± 1.07		
		**Median (IQR)**	9(5–10)		
	**UWS**	**Mean ± SD.**	0.21 ± 0.09		
		**Median (IQR)**	0.2(0.1–0.4)		

### Descriptive analysis of (SXI, CODS scores, and UWS) with different demographic and medical parameters

**Table 5:** Descriptive analysis of SXI and different demographic and medical parameters (*n* = 204)

Parameter	Item	No (%)	mean ± SD	(IQR)	Test of sig.	*P*-value
Age	**< 50**	71(35%)	11.77 ± 1.65	12(9–14)	T=-10.74	< 0.0001*
	**> 50**	133(65%)	13.53 ± 1.09	13(12–15)		
Gender	**Male**	109(53%)	11.94 ± 1.33	12(9–14)	T=-3.95	< 0.0001*
	**Female**	95(47%)	14.04 ± 0.89	14(12–15)		
Educational level	**Low**	53(26%)	13.11 ± 1.53	13(9–15)	F = 0.577	0.578ns
	**Middle**	98(48%)	12.87 ± 1.59	13(9–15)		
	**High**	53(26%)	12.83 ± 1.49	13(9–15)		
primary Tumor	**Oral cavity**	142(70%)	13.69 ± 0.91	13(12–15)	F = 152.29	< 0.0001*
	**Oropharynx**	51(25%)	11.62 ± 0.85	12(10–13)		
	**Nasopharynx**	11(5%)	9.75 ± 1.3	9(9 − 0)		
	**Larynx**	0				
T-stage	**T1**	64(31%)	12.17 ± 2.11	13(9–15)	F = 51.20	< 0.0001*
	**T2**	105(51%)	12.8 ± 1.77	13(9–15)		
	**T3**	35(17%)	14.66 ± 2.62	15(9–15)		
N-stage	**N0**	28(14%)	11.64 ± 1.73	12(9–14)	F = 41.74	< 0.0001*
	**N1**	83(41%)	12.82 ± 1.68	13(9–15)		
	**N2**	93(46%)	13.40 ± 1.10	13(10–15)		
Histological grade	**Grade I**	53(22%)	12.39 ± 1.27	13(9–14)	F = 23.92	< 0.0001*
	**Grade II**	85(42%)	12.41 ± 1.58	13(9–15)		
	**Grade III**	35(17%)	13.47 ± 1.11	13(10–15)		
	**Grade IV**	31(15%)	14.66 ± 0.7	15(12–15)		
Medical History	**Hypertension**	54(26%)	13.11 ± 1.61	13(9–15)	F = 1.22	0.727ns
	**Hypothyroid**	14(7%)	12.14 ± 1.46	12.5(9–14)		
	**Free**	23(11%)	13 ± 1	13(11–15)		
	**Leukemia**	24(12%)	12.96 ± 1.27	13(9–15)		
	**Diabetes**	64(31%)	12.91 ± 1.72	13(9–15)		
	**Hypertension & diabetes**	19(9%)	13.21 ± 1.65	13(9–15)		
	**Cardiovascular disease**	6(3%)	11.83 ± 1.72	12(9–14)		
Cancer Family History	**Positive**	57(28%)	12.72 ± 1.11	13(9–15)	T=-0.253	0.23ns
	**Negative**	147(72%)	13 ± 1.06	13(9–15)		
CRT	**Yes**	181(89%)	13.22 ± 1.29	13(9–15)	T = 7.76	< 0.0001*
	**No**	23(11%)	10.57 ± 1.41	11(9–13)		

SD: Standard deviationt: Student t-testF: F for One way ANOVA test

p: p value for Relation between SXI score and different parameters

*: Statistically significant at *p* ≤ 0.05 ns: non significant

Factors affecting SXI scores as highlighted in Table 5:

**Age**: Older patients (> 50 years) also showed higher SXI values (13.5 ± 1.1) compared with younger patients (11.8 ± 1.7, *p* < 0.001). **Gender**: Women reported significantly higher xerostomia scores than men (14.0 ± 0.9 vs. 11.9 ± 1.3, *p* < 0.001). **Primary Tumor Location**: SXI scores were highest in patients with tumors of the oral cavity (13.69) and lowest in those with tumors of the nasopharynx (9.75, *p* < 0.0001). **T-Stage**,** N-Stage**,** and Histological Grade**: Higher SXI scores were linked to higher-grade tumors (Grade IV) and advanced tumor stages (T3, N2) (*p* < 0.0001). **Chemoradiotherapy (CRT)**: SXI scores were higher (13.22) for CRT patients than for non-CRT patients (10.57, *p* < 0.0001). Therefore, Age, gender, tumor characteristics, and CRT strongly influence subjective xerostomia severity.

Factors affecting CODS scores as highlighted in Table 6:

**Table 6:** Descriptive analysis of **CODS** and different demographic and medical parameters (*n* = 204)

Parameter	Item	No (%)	mean ± SD	(IQR)	Test of sig.	*P*-value
Age	**< 50**	71(35%)	8.01 ± 1.06	8(5–9)	T=-8.06	< 0.0001*
	**> 50**	133(65%)	9.25 ± 0.53	9(8–10)		
Gender	**Male**	109(53%)	8.45 ± 1.10	9(7–10)	T=-13.28	< 0.0001*
	**Female**	95(47%)	9.02 ± 0.97	9(8–10)		
Educational level	**Low**	53(26%)	8.85 ± 0.95	9(7–10)	F = 0.549	0.563ns
	**Middle**	98(48%)	8.65 ± 1.13	9(7–10)		
	**High**	53(26%)	8.70 ± 1.1	9(8–10)		
primary Tumor	**Oral cavity**	142(70%)	9.13 ± 0.63	9(8–10)	F = 220.46	< 0.0001*
	**Oropharynx**	51(25%)	8.22 ± 0.83	8(7–9)		
	**Nasopharynx**	11(5%)	5.64 ± 0.5			
	**Larynx**	0				
T-stage	**T1**	64(31%)	7.95 ± 1.10	8(8–10)	F = 41.07	< 0.0001*
	**T2**	105(51%)	8.82 ± 0.77	9(7–10)		
	**T3**	35(17%)	9.8 ± 0.72	10(9–10)		
N-stage	**N0**	28(14%)	7.50 ± 1.37	8(8–9)	F = 16.02	< 0.0001*
	**N1**	83(41%)	8.54 ± 1.02	9(7–10)		
	**N2**	93(46%)	9.24 ± 0.58	9(8–10)		
Histological grade	**Grade I**	53(22%)	8.32 ± 0.92	9(7–9)	F = 28.95	< 0.0001*
	**Grade II**	85(42%)	8.4 ± 1.17	9(8–10)		
	**Grade III**	35(17%)	9.06 ± 0.58	9(8–10)		
	**Grade IV**	31(15%)	9.88 ± 0.34	10(9–10)		
Medical History	**Hypertension**	54(26%)	8.94 ± 0.93	9(8–10)	F = 1.34	0.299ns
	**Hypothyroid**	14(7%)	8.36 ± 1.04	9(8–9)		
	**Free**	23(11%)	8.74 ± 0.5	9(8–10)		
	**Leukemia**	24(12%)	8.58 ± 1.11	9(8–10)		
	**Diabetes**	64(31%)	8.63 ± 1.23	9(7–10)		
	**Hypertension & diabetes**	19(9%)	8.95 ± 0.94	9(8–10)		
	**Cardiovascular disease**	6(3%)	8.17 ± 1.17	9(8–9)		
Cancer Family History	**Positive**	57(28%)	8.68 ± 1.06	9(8–10)	F=-1.20	0.801ns
	**Negative**	147(72%)	8.73 ± 1.12	9(7–10)		
CRT	**Yes**	178 (98%)	8.95 ± 0.79	9(7–10)	T = 8.42	< 0.0001*
	**No**	23(12%)	8.01 ± 1.06	8(8–9)		

SD: Standard deviation, t: Student t-test, F: F for One way ANOVA test

p: p value for Relation between SXI score and different parameters

*: Statistically significant at *p* ≤ 0.05 ns: non-significant

**Age**: Patients over 50 years demonstrated more severe clinical dryness (9.3 ± 0.5 vs. 8.0 ± 1.1, *p* < 0.001). **Gender**: Similarly, CODS scores were higher in women than in men (9.0 ± 1.0 vs. 8.5 ± 1.1, *p* < 0.001). **Primary Tumor Location**: Tumors in the nasopharynx had the lowest CODS scores (5.64, *p* < 0.0001), whereas those in the oral cavity were the highest (9.13). **Histological Grade**,** T-Stage**,** and N-Stage**: Higher-grade tumors (Grade IV) and advanced tumor stages (T3, N2) had higher CODS scores (*p* < 0.0001). **CRT**: Patients who received CRT had higher CODS (8.95) than those who did not (8.01, *p* < 0.0001). The findings of CODS are consistent with those of SXI, suggesting that oral dryness is influenced by tumor development and CRT.

Factors affecting UWS as highlighted in Table 7:

**Table 7:** Descriptive analysis of **UWS** and different demographic and medical parameters. (*n* = 204)

Parameter	Item	No (%)	mean ± SD	(IQR)	Test of sig.	*P*-value
Age	**< 50**	66(35%)	0.27 ± 0.1	0.3(0.1–0.4)	T = 7.88	< 0.0001*
	**> 50**	123(65%)	0.17 ± 0.06	0.2(0.1–0.3)		
Gender	**Male**	97(51%)	0.15 ± 0.07	0.2(0.1–0.4)	T = 9.56	< 0.0001*
	**Female**	92(49%)	0.25 ± 0.08	0.1(0.1–0.4)		
Educational level	**Low**	50(27%)	8.85 ± 0.94	0.2(0.1–0.4)	F = 0.620	0.539ns
	**Middle**	91(48%)	8.65 ± 1.13	0.2(0.1–0.4)		
	**High**	48(25%)	8.7 ± 1.09	0.2(0.1–0.4)		
primary Tumor	**Oral cavity**	142(75%)	0.16 ± 0.05	0.2(0.1–0.2)	F = 220.45	< 0.0001*
	**Oropharynx**	47(25%)	0.38 ± 0.04	0.3(0.2–0.4)		
	**Nasopharynx**	0	0.29 ± 0.06	0.4(0.4-0)		
	**Larynx**	0				
T-stage	**T1**	64(31%)	0.25 ± 0.1	0.2(0.1–0.4)	F = 40.28	< 0.0001*
	**T2**	105(51%)	0.21 ± 0.07	0.2(0.1–0.4)		
	**T3**	35(17%)	0.11 ± 0.05	0.1(0.1–0.4)		
N-stage	**N0**	20(11%)	0.29 ± 0.10	0.3(0.1–0.4)	F = 25.76	< 0.0001*
	**N1**	77(41%)	0.22 ± 0.10	0.2(0.1–0.4)		
	**N2**	92(49%)	0.17 ± 0.05	0.2(0.1–0.4)		
Histological grade	**Grade I**	49(26%)	0.24 ± 0.08	0.2(0.1–0.4)	F = 25.9	< 0.0001*
	**Grade II**	74(39%)	0.23 ± 0.09	0.2(0.1–0.4)		
	**Grade III**	35(19%)	0.18 ± 0.06	0.2(0.1–0.3)		
	**Grade IV**	31(16%)	0.11 ± 0.04	0.1(0.1–0.2)		
Medical History	**Hypertension**	51(27%)	0.2 ± 0.09	0.2(0.1–0.4)	F = 0.831	0.576ns
	**Hypothyroid**	13(7%)	0.25 ± 0.08	0.2(0.1–0.4)		
	**Free**	22(12%)	0.2 ± 0.07	0.2(0.1–0.4)		
	**Leukemia**	22(12%)	0.2 ± 0.07	0.2(0.1–0.4)		
	**Diabetes**	85(31%)	0.21 ± 0.1	(0.1–0.4)		
	**Hypertension & diabetes**	18(10%)	0.19 ± 0.08	0.2(0.1–0.4)		
	**Cardiovascular disease**	5(3%)	0.25 ± 0.1	0.25(0.1–0.4)		
Cancer Family History	**Positive**	52(28%)	0.22 ± 0.08	0.2(0.1–0.4)	T = 1.021	0.309ns
	**Negative**	137(72%)	0.2 ± 0.09	0.2(0.1–0.4)		
CRT	**Yes**	178(94%)	0.19 ± 0.07	0.2(0.1–0.4)	T=-15.66	< 0.0001*
	**No**	11(6%)	0.37 ± 0.05	0.4(0.3–0.4)		

SD: Standard deviation, t: Student t-test, F: F for One way ANOVA test

p: p value for Relation between SXI score and different parameters

*: Statistically significant at *p* ≤ 0.05 ns: non-significant

**Age**: Older patients (> 50 years) also showed reduced UWS compared with younger patients (0.17 ± 0.06 vs. 0.27 ± 0.10 ml/min, *p* < 0.001). **Gender**: Men demonstrated lower UWS values (0.15 ± 0.07 ml/min) compared with women (0.25 ± 0.08 ml/min), highlighting a gender-related difference in objective salivary flow. **Primary Tumor Location**: Tumors of the oropharynx had higher UWS values (0.38 mL, *p* < 0.0001), whereas tumors of the oral cavity had the lowest values (0.16 mL). **T-Stage**,** N-Stage**,** and Histological Grade**: Lower UWS values (*p* < 0.0001) were associated with higher-grade tumors (Grade IV) and advanced tumor stages (T3, N2). **CRT**: Compared to patients who did not get CRT, those who did had a reduced UWS (0.19 mL, *p* < 0.0001).

### Correlations with severity of xerostomia

The relationships between xerostomia severity and various parameters, measured using SXI, CODS, and UWS as presented in Table 8 were as follows:

**Table 8:** Correlations of different demographic and medical parameters with severity of xerostomia / hyposalivation

Parameter	SXI		CODS		UWS		
	*r* (95% CI)	*p*-value	*r* (95% CI)	*p*-value		*r* (95% CI)	*p*-value
Age	0.222 (0.103–0.334)	0.0003*	0.387(0.2790.486)	< 0.0001*		−0.574 (− 0.659, − 0.474)	< 0.0001*
Gender	0.337(0.222,0.451)	< 0.0001*	0.359(0.245,0.473)	< 0.0001*		0.556 (0.453, 0.644.)	< 0.0001*
Educational level	0.118( -0.003,0.239)	0.057ns	0.087(-0.034,0.209)	0.159ns		0.053 (-0.085, 0.189)	0.450 ns
primary Tumor	0.894(0.866,0.916)	< 0.001*	0.894(0.812,0.880)	< 0.001*		-0.790 (-0.837, -0.733)	< 0.001*
T-stage	0.226 (0.108,0.338)	0.00023*	0.282 (0.166,0.390)	< 0.0001*		−0.509 (− 0.604, − 0.399)	< 0.0001*
N-stage	0.157(-0.120,0.123)	0.011*	0.266(-0.114,0.117)	0.011*		−0.444 (− 0.548,−0.327)	< 0.0001*
Histological grade	0.046 (-0.084,0.175)	0.488ns	0.072(-0.058,0.200)	0.276ns		-0.487 (0.194, 0.218)	< 0.0001*
Medical History	-0.0787 (-0.1982, 0.0431)	0.205ns	-0.0909 (-0.2100, 0.0308)	0.143ns		-0.0787(-0.1982, 0.0431)	0.205ns
Cancer Family History	-0.0917 (-0.736, 0.553)	0.780ns	-0.1478 (-0.742, 0.447)	0.626ns		-0.0917 (-0.736, 0.553)	0.780ns
CRT	0.795 (0.746, 0.836)	< 0.0001*	0.873(0.840, 0.899)	< 0.0001*		−0.635 (− 0.710, − 0.54)	< 0.0001*
Radi. fract.	0.2546(0.1375, 0.3648)	< 0.0001*	0.3980(0.2906, 0.4955)	< 0.0001*		−0.537 (-0.399 − 0.399)	< 0.0001*
total rad. Dose	0.293(0.177, 0.400)	< 0.0001*	0.405(0.298, 0.502)	< 0.0001*		−0.5468 (− 0.6364,−0.4427)	< 0.0001*

CI: Confidence interval, * significant (*p* < 0.05). ns: non-significant

1. **Age**: *r* = 0.222, *p* = 0.0003, and *r* = 0.387, *p* < 0.0001 correlated positively with SXI and CODS. The correlation with UWS is negative (*r* = -0.574, *p* < 0.0001). Poorer xerostomia symptoms (higher SXI and CODS) and decreased salivary flow (lower UWS) are associated with aging.
2. **Gender**: Significant association between UWS, CODS, and SXI. Subjective (SXI: *r* = 0.337) and clinical dryness (CODS: *r* = 0.359) showed positive correlations, while UWS showed negative correlations (*r* = -0.556, *p* < 0.0001). Females had decreased saliva production and worse subjective and clinical xerostomia.
3. **Location of the Primary Tumor**: A strong positive correlation with CODS (*r* = 0.894, *p* < 0.001) and SXI (*r* = 0.894). A negative correlation (*r* = -0.790, *p* < 0.001) with UWS was observed. Oral cavity cancers are associated with worse outcomes; tumor placement has a substantial impact on the severity of xerostomia.
4. **Radiotherapy**,** T-Stage**,** and N-Stage**: Higher SXI and CODS and lower UWS are correlated with higher tumor stages (T and N) and greater radiation doses (all *p* < 0.0001). CRT: strongly associated with UWS (*r* = -0.635, *p* < 0.0001), SXI (*r* = 0.795), and CODS (*r* = 0.873).

### Regression models of xerostomia and hyposalivation

As shown in Table 9, when the Summated Xerostomia Inventory (SXI) was employed as the outcome, several predictors emerged as significant. Older age increased the odds of xerostomia by 6% per year (OR = 1.06, 95% CI: 1.03–1.09, *p* < 0.001). Female gender was strongly associated with worse symptoms, with females experiencing between 4- and 33-fold higher odds compared with males (OR = 8.7, 95% CI: 4.03–33.09, *p* < 0.001). Tumor stage also influenced risk, with advanced T-stage (OR = 2.43, 95% CI: 1.39–4.38, *p* < 0.001) and N-stage (OR = 1.72, 95% CI: 1.16–2.56, *p* = 0.007) both predicting more severe xerostomia. Importantly, treatment-related variables were significant: every 10 Gy increase in radiation dose raised the odds of xerostomia by 13% (OR = 1.13 per 10 Gy, 95% CI: 1.08–1.19, *p* < 0.001), and each additional fraction increased the odds by 23% (OR = 1.23, 95% CI: 1.11–1.37, *p* < 0.001). In contrast, histological grade and primary tumor site were not significant predictors.

**Table 9:** Regression model prediction Xerostomia if **SXI** is 9 or above

Term	Odds Ratio (OR)	95% CI	Test Statistic	*p*-value
Age	1.06	1.03–1.09	3.49	< 0.001*
Gender (female vs. male)	8.7	4.03–33.09	4.55	< 0.001*
Educational level	4.82	2.50–9.20	4.75	< 0.001*
Primary tumor	3.42	0.00 – ∞	0.65	0.997 ns
T-stage	2.43	1.39–4.38	3.51	< 0.001*
N-stage	1.72	1.16–2.56	2.69	0.007*
Histological grade	1.54	0.77–3.08	1.76	0.079 ns
Medical history	1.17	0.97–1.40	1.65	0.099 ns
Cancer family history	0.90	0.41–1.91	-0.25	0.800 ns
CRT	1.10	0.00 – ∞	0.01	0.994 ns
Radiation fractions	1.23	1.11–1.37	3.91	< 0.001*
Total radiation dose (per Gy)	1.0013	1.001–1.002	4.38	< 0.0001*

CI: confidence interval, * significant (*p* < 0.05). ns: non-significant

Table 10 presents the regression model using the Clinical Oral Dryness Score (CODS) as the outcome. Similar to SXI, older age increased the odds of severe dryness by ~ 10% per year (OR = 1.10, 95% CI: 1.07–1.14, *p* < 0.0001). Female patients again exhibited greater xerostomia severity (OR = 0.11, 95% CI: 0.05–0.26, *p* < 0.0001, with males relatively protected). Disease stage remained important, as advanced T-stage (OR = 12.36, 95% CI: 1.03–47.9, *p* = 0.001) and N-stage (OR = 2.37, 95% CI: 1.62–3.49, *p* < 0.0001) both increased xerostomia severity. Additional predictors included histological grade (OR = 1.55, 95% CI: 1.03–2.32, *p* = 0.002), family history of cancer (OR = 10.4, 95% CI: 4.15–26.0, *p* < 0.0001), and concurrent chemoradiotherapy (CRT), which raised the odds nearly sixfold (OR = 5.92, 95% CI: 4.61–7.23, *p* < 0.0001). Radiation dose and fractionation also showed strong dose–response effects, with ~ 10% higher odds of xerostomia per 10 Gy increase (OR = 1.10, 95% CI: 1.001–1.002, *p* < 0.0001) and 39% higher odds with each additional fraction (OR = 1.39, 95% CI: 1.24–1.56, *p* < 0.0001). Educational level and primary tumor site did not significantly influence CODS.

**Table 10:** Regression model prediction Hyposalivation if **CODS **is 0-10

Term	Odds Ratio (OR)	95% CI	TestStatistic	p-value
Age (per year)	1.10	1.07 – 1.14	5.83	<0.0001*
Gender (female vs. male)	0.11	0.05 – 0.26	-5.14	<0.0001*
Educational level	1.12	0.72 – 1.94	0.26	0.795 ns
Primary tumor	1.8 × 10¹⁶	0.00 – ∞	-0.58	0.736 ns
T-stage	12.36	1.03 – 47.9	3.32	0.001*
N-stage	2.37	1.62 – 3.49	4.40	<0.0001*
Histological grade	1.55	1.03 – 2.32	3.12	0.002*
Medical history	1.10	0.74 – 1.66	1.39	0.889 ns
Cancer family history	10.4	4.15 – 26.0	5.00	<0.0001*
CRT (yes vs. no)	5.92	4.61 – 7.23	8.88	<0.0001*
Radiation fractions	1.39	1.24 – 1.56	5.69	<0.0001*
Total radiation dose (per Gy)	1.001	1.001 – 1.002	5.63	<0.0001*

CI: confidence interval, * significant (p<0.05). ns: non-significant

Finally, Table 11 examined predictors of unstimulated salivary flow rate (UWS ≤ 0.4 ml/min). Older age (OR = 1.06, 95% CI: 1.03–1.09, *p* < 0.001), female gender (OR = 0.24, 95% CI: 0.03–0.25, *p* < 0.001, with males relatively protected), and advanced T-stage (OR = 2.43, 95% CI: 1.48–3.99, *p* < 0.001) and N-stage (OR = 1.72, 95% CI: 1.16–2.57, *p* = 0.007) were all associated with lower salivary output. Histological grade (OR = 1.54, 95% CI: 1.09–2.17, *p* = 0.014) and comorbid medical conditions (OR = 1.33, 95% CI: 1.01–1.65, *p* < 0.0001) also predicted hyposalivation. Radiation parameters were again critical: each additional fraction increased the odds of hyposalivation by ~ 23% (OR = 1.23, 95% CI: 1.10–1.31, *p* < 0.001), and every 10 Gy increase in dose increased the odds by ~ 13% (OR = 1.13 per 10 Gy, 95% CI: 1.001–1.002, *p* < 0.001). In this model, CRT and family history of cancer were not significant predictors.

**Table 11:** Regression model prediction hyposalivation if **UWS** is 0.4 ml or below

Term	Odds Ratio (OR)	95% CI	Test Statistic	*p*-value
Age (per year)	1.06	1.03–1.09	3.49	< 0.001*
Gender (female vs. male)	0.24	0.03–0.25	-4.55	< 0.001*
Educational level	0.87	0.30–2.48	-0.65	0.514 ns
Primary tumor	1.65	0.74–3.82	1.21	0.227 ns
T-stage	2.43	1.48–3.99	3.51	< 0.001*
N-stage	1.72	1.16–2.57	2.69	0.007*
Histological grade	1.54	1.09–2.17	2.46	0.014*
Medical history (present vs. none)	1.33	1.01–1.65	8.18	< 0.0001*
Cancer family history	0.91	0.48–1.74	-0.28	0.780 ns
CRT (yes vs. no)	1.00	0.00 – ∞	0.01	0.994 ns
Radiation fractions	1.23	1.10–1.31	3.91	< 0.001*
Total radiation dose (per Gy)	1.0013	1.001–1.002	4.38	< 0.001*

CI: confidence interval, * significant (*p* < 0.05). ns: non-significant

**Fig. 2:**
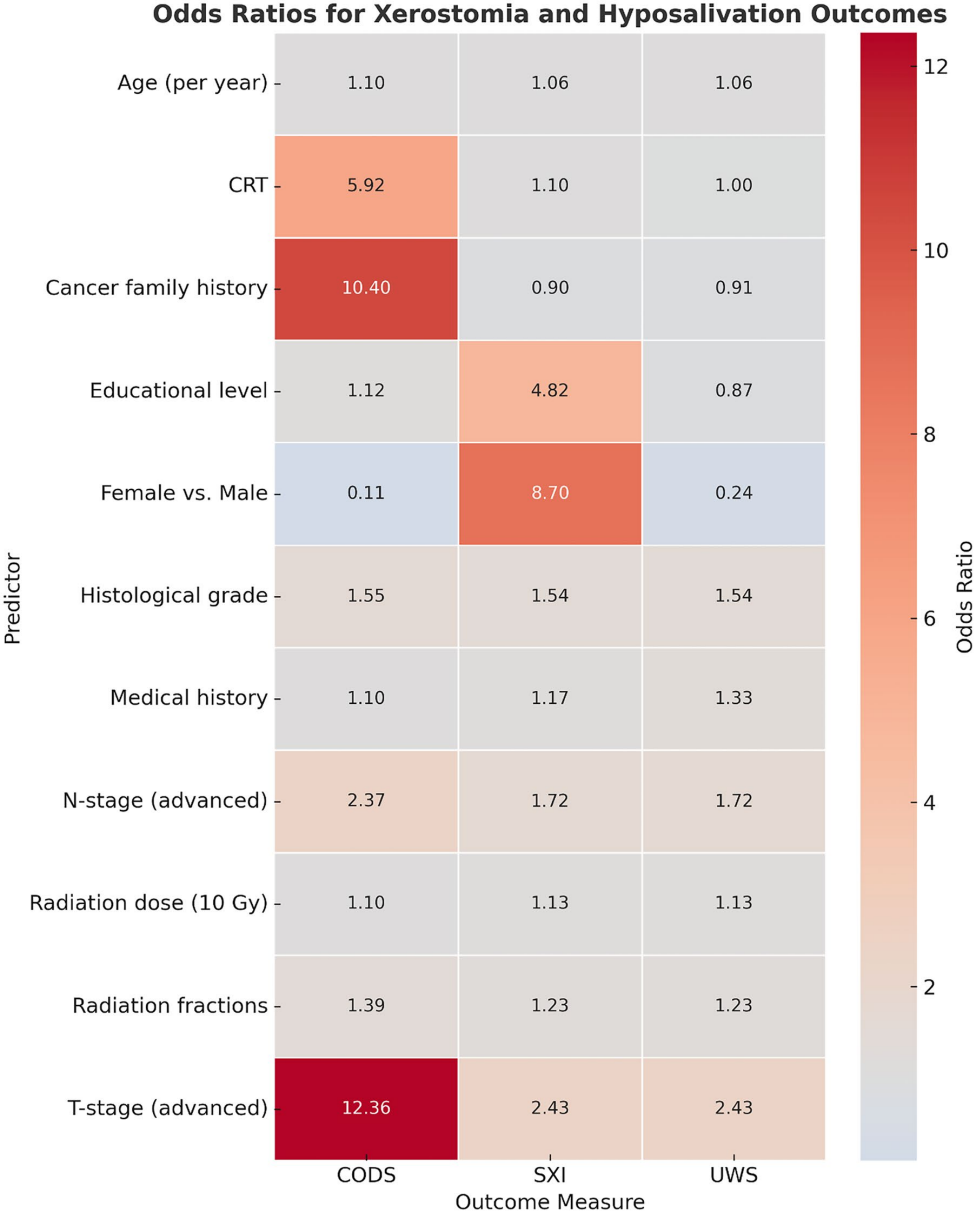
Heatmap of odds ratios (OR) for predictors of xerostomia across SXI, CODS, and UWS

In summary, across all three models (SXI, CODS, UWS), older age, female gender, advanced tumor stage, and higher radiation dose/fraction consistently predicted worse xerostomia and hyposalivation. CODS models additionally implicated histological grade, family history, and CRT, while UWS models highlighted comorbid medical conditions. Together, these findings demonstrate that both patient-related and treatment-related factors strongly influence late salivary dysfunction, with radiation dose and fractionation emerging as central modifiable determinants (Fig. 2).

## Discussion

Head and neck cancer (HNC) is commonly treated with radiotherapy, either alone or in combination with chemotherapy and surgery [29]. Studies indicate that over 80% of HNC patients experience xerostomia and salivary gland hypofunction following RT [30].

### Prevalence of xerostomia and the Egyptian context

In our multicentric cross-sectional study of 261 Egyptian HNC survivors, we found that late xerostomia was (78%), which is a notably high prevalence, and that its severity was influenced by a complex interaction of treatment-related, tumor-related, and demographic factors. This was in similar to previous studies ranging from 64% to 91% [31–34].

In the current study, unstimulated salivary flow rates (UWS) showed a median of 0.2 mL, indicating a significant reduction in saliva production. This finding is consistent with previous studies that reported low unstimulated salivary flow rates in HNC patients following RT [35–38].

This increased burden needs to be recognized in the context of Egypt’s unique clinical environment rather than being solely attributable to treatment factors. A significant factor is the high percentage of patients in our cohort who present with advanced tumor stages (T3/T4, N2), which is a result of the regional healthcare system’s often delayed diagnosis, and restricted IMRT access.

Additionally, socioeconomic barriers prevent access to advanced radiotherapy techniques and long-term palliative care, while cultural dietary practices exacerbate the functional impairment of dry mouth. To address this disproportionate severity, this combination of factors calls for the creation of easily accessible, reasonably priced, and culturally sensitive management protocols.

### Impact of sociodemographic factors

Regarding the demographic data, the results showed a male predominance among those with xerostomia complaint (53%), while females were (47%). This was in line with previous studies [36, 37, 39–41].

This may be attributed to higher rates of alcohol consumption, tobacco use, and prolonged sun exposure in males. Additionally, men tend to visit dentists less frequently than women and are more likely to seek dental care for urgent issues rather than preventive care. In contrast, women exhibit better oral health behaviors, greater oral health knowledge, and more positive attitudes toward dental visits [42].

Although the prevalence of cancer is higher in men, females reporting significantly higher xerostomia scores (14.0 ± 0.9 vs. 11.9 ± 1.3, *p* < 0.001) and lower UWS values than men.

Patients over 50 years old had the highest prevalence of xerostomia, which is consistent with known evidence of age-related dysfunction of the salivary glands. This finding aligns with previous research conducted in various parts of the world [37, 43, 44]. Conversely, other studies have suggested that xerostomia may be more common in females [17, 45]. These disparities most likely result from differences in risk factor reporting and study methodologies.

The severity of xerostomia does not appear to be significantly influenced by the level of education (*p* = 0.578) in the current study. However, education level may serve as a proxy for socioeconomic status, which could indirectly affect treatment compliance or access to healthcare. Additionally, 88% of HNC patients in this study had various medical conditions, indicating the presence of underlying potential risk factors for cancer development.

### Impact of tumor-related factors

Tumor size plays a critical role in radiation planning and, consequently, in salivary gland exposure. Our study observed that xerostomia severity increased with advancing T-stage, with T3 patients exhibiting the highest SXI and CODS scores, which was consistent with findings from **Schulz et al.**,** 2021** [46]. Similarly, as histologic grades worsened, xerostomia severity increased, with Grade IV tumors having the highest SXI and CODS scores, suggesting a connection between tumor aggressiveness and salivary gland susceptibility.

The oral cavity was the most common primary tumor location (54%) in our study, which is consistent with previous research [37, 47, 48] reporting a higher prevalence of xerostomia among patients with oral cavity tumors. Since radiation directly affects the salivary glands in the region, this could explain the high incidence of xerostomia. However, the significantly lower SXI and CODS scores observed in individuals with oropharyngeal tumors suggest the need for further investigation into potential protective factors or reduced salivary gland exposure in these cases.

### Effects of treatment-related elements

Compared to patients receiving radiotherapy alone, those who underwent CRT exhibited significantly worse xerostomia measures (SXI, CODS, and UWS). This highlights the need for strategies to minimize salivary gland exposure in this subgroup and further establishes CRT as a major factor contributing to xerostomia severity. Additionally, our study’s crude analysis revealed a strong correlation between RT, CT, and the decline in stimulated salivary flow, which aligns with previous research [37, 46]. However, since many prior studies did not measure unsimulated salivary flow rates, they primarily found a statistically significant correlation between RT, CT, and subjective xerostomia symptoms [49, 50].

According to **Hey et al. (2009)**, patients undergoing RT + CT tolerated radiation doses that were 7–8 Gy lower than those who received only radiation. These findings indicate that radiation therapy combined with concurrent chemotherapy has a higher propensity to damage parotid gland tissue [51]. Chemotherapy can cause myelosuppression because it destroys or inhibits cell proliferation without distinguishing between cancerous and healthy cells. Additionally, chemotherapy negatively impacts the salivary glands [52–54].

Regarding radiotherapy dosage, patients with xerostomia received a slightly higher mean radiation dose (6496 ± 394 cGy) compared to the mean total dose for all patients (6420.31 ± 489.99 cGy). Correlation analysis demonstrated a significant relationship between total radiation exposure and the severity of xerostomia across all indices (SXI, CODS, and UWS), with p-values < 0.0001 and positive correlation coefficients, aligning with previous studies [12, 55, 56]. The regression analysis further highlighted this effect, as a total radiation dose odds ratio of 1.0013 (*p* < 0.0001) indicated that even slight dose increases significantly raised the likelihood of xerostomia.

For radiotherapy fractionation, the mean number of fractions given to patients with xerostomia was slightly higher (32.2 ± 2.26) compared to the overall patient group (31.87 ± 2.64). Correlation analysis confirmed a significant association between the number of fractions and the occurrence of xerostomia. Additionally, regression analysis identified fractions as a significant predictor, with an odds ratio of 1.393 (*p* < 0.0001), indicating that the probability of xerostomia increases significantly with each additional fraction.

The results indicate that xerostomia prevalence increases when dose and fractionation thresholds are exceeded. Future research should explore optimal fractionation and dosage schedules to minimize xerostomia while maintaining effective tumor control. Additionally, longitudinal studies are required to determine whether salivary gland function partially recovers after radiation therapy or whether xerostomia persists in patients receiving higher doses or fractions.

The necessity for treatment planning that balances tumor management and salivary gland preservation is underscored by the significant correlation between radiation dose, fractionation, and xerostomia severity. Implementing gland-sparing techniques such as intensity-modulated radiotherapy (IMRT), hypofractionation, or radioprotectants could significantly improve the quality of life for patients by reducing the severity of xerostomia.

### Comparison to previous studies

Our results are consistent with the body of research demonstrating that, older age, female sex, advanced tumor stage, higher radiation dose, and chemotherapy have been identified as common risk factors for xerostomia in previous reports [22, 48, 57]. Additionally, this study highlights the significant influence of primary tumor location, a variable that has received little attention before, as demonstrated by its strong correlation with oral cavity tumors.

However, in a study by **Schulz et al.**,** 2021** [46], hyposalivation was not associated with age, sex, or primary tumor location, suggesting some discrepancies in risk factor associations. This disparity could be explained by variations in patient groups, salivary output measurement methods, or the particular statistical models used.

### Clinical implications

These findings emphasize the importance of integrating xerostomia management into treatment plans for head and neck cancer. A stepped management strategy is advised for xerostomia that has been established. This includes using moisturizing gels and saliva substitutes as a first line of treatment for symptoms, moving on to pharmacological stimulants like pilocarpine for patients who qualify, and non-pharmacological options like acupuncture. Notably, natural agents such as Manuka honey [27] and thyme honey [26] have emerged a complementary, safe, effective therapies for xerostomia, as these agents have shown significant improvement in both subjective and objective xerostomia measures in randomized trials without causing negative side effects. Preventive measures, such as intensive preventive oral care programs and pre-treatment dental evaluations, are crucial for high-risk individuals in order to minimize long-term consequences. To greatly enhance patients’ quality of life, survivorship care must incorporate this individualized, step-by-step method.

## Conclusions

This study highlights a high prevalence (78%) of xerostomia and hyposalivation in HNC patients following RT), with or without CT. The findings indicate that key treatment-related factors, such as fractionation regimens and total radiation dose, are strongly associated with the severity of xerostomia and hyposalivation. Additionally, CT further impairs salivary gland function, leading to long-term complications such as reduced unstimulated salivary flow and poor oral health. Given the strong correlation between xerostomia severity and increased radiation exposure, innovative treatment approaches are necessary to minimize glandular damage. Similarly, the observed associations with factors such as age, tumor location, and chemotherapy underscore the importance of patient-specific risk assessments in treatment planning.

### Limitations

The findings may not be fully generalizable to broader populations with varied demographics or treatment approaches, as the study cohort may not entirely reflect the diversity of head and neck cancer patients. Furthermore, since only patients with subjective xerostomia complaints were further evaluated, there is a potential **selection bias** that may underestimate the prevalence of asymptomatic hyposalivation. Despite the use of validated instruments, self-reported xerostomia indices (such as the SXI) are inherently subjective, which may introduce reporting bias. Finally, long-term recovery trajectories and changes in salivary gland function over time were not assessed, as the study focused on post-treatment outcomes.

## Data Availability

Research data supporting this publication is available from the corresponding author upon request.

## Footnotes

1 Schulz, Riéli Elis, et al. “Prevalence of hyposalivation and associated factors in survivors of head and neck cancer treated with radiotherapy.” Journal of Applied Oral Science 29 (2021): e20200854.

2 R Core Team (2024). R: A language and environment for statistical computing. R Foundation for Statistical Computing, Vienna, Austria. URL https://www.R-project.org/.

3 R Core Team (2024). R: A language and environment for statistical computing. R Foundation for Statistical Computing, Vienna, Austria. URL https://www.R-project.org/.
